# Comparative analysis of deep learning and radiomic signatures for overall survival prediction in recurrent high-grade glioma treated with immunotherapy

**DOI:** 10.1186/s40644-024-00818-0

**Published:** 2025-01-21

**Authors:** Qi Wan, Clifford Lindsay, Chenxi Zhang, Jisoo Kim, Xin Chen, Jing Li, Raymond Y. Huang, David A. Reardon, Geoffrey S. Young, Lei Qin

**Affiliations:** 1 Department of Radiology, the Key Laboratory of Advanced Interdisciplinary Studies Center, the First Affiliated Hospital of Guangzhou Medical University, Guangzhou, China; 2 Department of Imaging, Dana-Farber Cancer Institute, Harvard Medical School, Boston, MA USA; 3 Department of Radiology, Division of Biomedical Imaging and Bioengineering, UMass Chan Medical School, Worcester, MA USA; 4 Digital Medical Research Center, School of Basic Medical Sciences, Fudan University, Shanghai, China; 5 Department of Radiology, Brigham and Women’s Hospital, Harvard Medical School, Boston, MA USA; 6 Guangzhou First People’s Hospital, School of Medicine, South China University of Technology, Guangzhou, Guangdong China; 7 Department of Radiology, the Affiliated Cancer Hospital of Zhengzhou University (Henan Cancer Hospital), Zhengzhou, China; 8 Center for Neuro-Oncology, Dana-Farber Cancer Institute, Harvard Medical School, Boston, MA USA

**Keywords:** High-grade glioma, Convolutional neural networks, Deep learning, Radiomics, Overall survival

## Abstract

**Background:**

Radiomic analysis of quantitative features extracted from segmented medical images can be used for predictive modeling of prognosis in brain tumor patients. Manual segmentation of the tumor components is time-consuming and poses significant reproducibility issues. We compare the prediction of overall survival (OS) in recurrent high-grade glioma(HGG) patients undergoing immunotherapy, using deep learning (DL) classification networks along with radiomic signatures derived from manual and convolutional neural networks (CNN) automated segmentation.

**Materials and methods:**

We retrospectively retrieved 154 cases of recurrent HGG from multiple centers. Tumor segmentation was performed by expert radiologists and a convolutional neural network (CNN). From the segmented tumors, 2553 radiomic features were extracted for each case. A robust feature subset was selected using intraclass correlation coefficient analysis between manual and automated segmentations. The data was divided into a 9:1 ratio and validated through ten-fold cross-validation and tested on a rotating test set. Features selection was done by the Kruskal–Wallis test. The Radiomics-based OS predictions, generated using Support Vector Machine (SVM), were compared between the two segmentation approaches and against OS prediction by the CNN model adapted for classification. Model efficacy was evaluated using the area under the receiver operating characteristic curve (AUC).

**Results:**

The clinical model AUC for OS prediction was 0.640 ± 0.013 (mean ± 95% confidence interval) in the training set and 0.610 ± 0.131 in the test set. The radiomics prediction of OS based on manual segmentation outperformed automatic segmentation (AUC of 0.662 ± 0.122 vs. 0.471 ± 0.086, respectively) in the test set. Robust features improved the performance of manual segmentation to AUC of 0.700 ± 0.102, of automated segmentation to 0.554 ± 0.085. The CNN prognosis model demonstrated promising results, with an average AUC of 0.755 ± 0.071 for training sets and 0.700 ± 0.101 for the test set.

**Conclusion:**

Manual segmentation-derived radiomic features outperformed automated segmentation-derived features for predicting OS in recurrent high-grade glioma patients undergoing immunotherapy. The end-to-end CNN prognosis model performed similarly to radiomics modeling using manual-segmentation-derived features without the need for segmentation. The potential time-saving must be weighed against the lower interpretability of end-to-end black box modeling.

## Introduction

High-grade gliomas (HGG) represent the most common and aggressive infiltrative glioma in adults, characterized by rapid growth and high invasiveness, with glioblastoma (GBM) being the most common type [1]. The prognosis for patients with HGG is universally poor, despite advancements in standard care, including maximal surgical resection, concurrent chemoradiotherapy, and maintenance chemotherapy [2, 3]. Immunotherapies are transforming the care of patients with melanoma and lung cancer. Although these therapies have not shown substantial benefit for HGG patients as a whole, data suggests that a subset of patients may respond [4]. Identifying these subsets of patients who may benefit from immunotherapy remains a critical goal.

Radiomics, the extraction and analysis of quantitative imaging features from brain tumor MRI, allows automated, quantitative, diagnostic and prognostic modeling [5, 6]. Feature extraction for radiomic analysis requires ‘tumor segmentation’, by annotating a region of interest (ROI) containing the tumor on brain MRI. Manual segmentation by radiologists is the gold standard, but it is both time-consuming and labor-intensive, hindering research use and precluding clinical translation of radiomics [7]. Deep-learning approaches that allow automated or annotation-independent methods could significantly accelerate the research and clinical application of radiomics.

In this study, we report the development and validation of two deep learning approaches, based on a convolutional neural network (CNN), designed to accelerate radiomics analysis of recurrent HGG patients undergoing immunotherapy: a CNN-segmentation model for automated image segmentation, and an end-to-end, CNN-prognosis model that inputs unsegmented MRI images and outputs a survival prediction. We compare the accuracy of patient overall survival (OS) prediction based on radiomic features obtained from the automated segmentation, compared to features extracted by manual segmentation, and compared to the accuracy of the CNN-prognosis model.

## Materials and methods

### Study participants

This retrospective medical records study was approved by our Institutional Review Board with waiver of informed consent, and conducted in compliance with HIPAA regulations. The patient cohort used in this study was part of our previous research [8]. 154 patients with histopathologically confirmed recurrent high-grade glioma who received immunotherapy between April 2014 and February 2019 were consecutively included. All patients received immunotherapy, with the majority undergoing anti-programmed death-ligand 1 (PD-L1) treatment (*n* = 151). A few patients received alternative agents, including anti-cluster of differentiation 137 (CD137; n = 1) and anti-lymphocyte activation gene 3 (LAG-3; relatlimab, *n* = 2) instead of anti-PD-L1. Many patients also received concurrent treatments alongside immunotherapy, including: Anti-cytotoxic T-lymphocyte–associated protein 4 (CTLA-4; ipilimumab, *n* = 1), anti-CD27 (varlilumab, *n* = 5), anti-colony-stimulating factor-1 (CSF-1; cabiralizumab, *n* = 1), anti-indoleamine 2,3-dioxygenase 1 (IDO-1; epacadostat, *n* = 4), anti-LAG-3 (*n* = 1), vaccines (*n* = 3), isocitrate dehydrogenase (IDH) inhibitor (*n* = 1),  bevacizumab (Avastin, *n* = 51), re-irradiation (*n* = 22), re-irradiation combined with Avastin (*n* = 7), re-irradiation combined with temozolomide (TMZ) (*n* = 6), gene therapy (Ad-RTS-12 veledimex, *n* = 2), carboplatin (*n* = 1), and gene-mediated cytotoxic immunotherapy (*n* = 1).

**Fig. 1:**
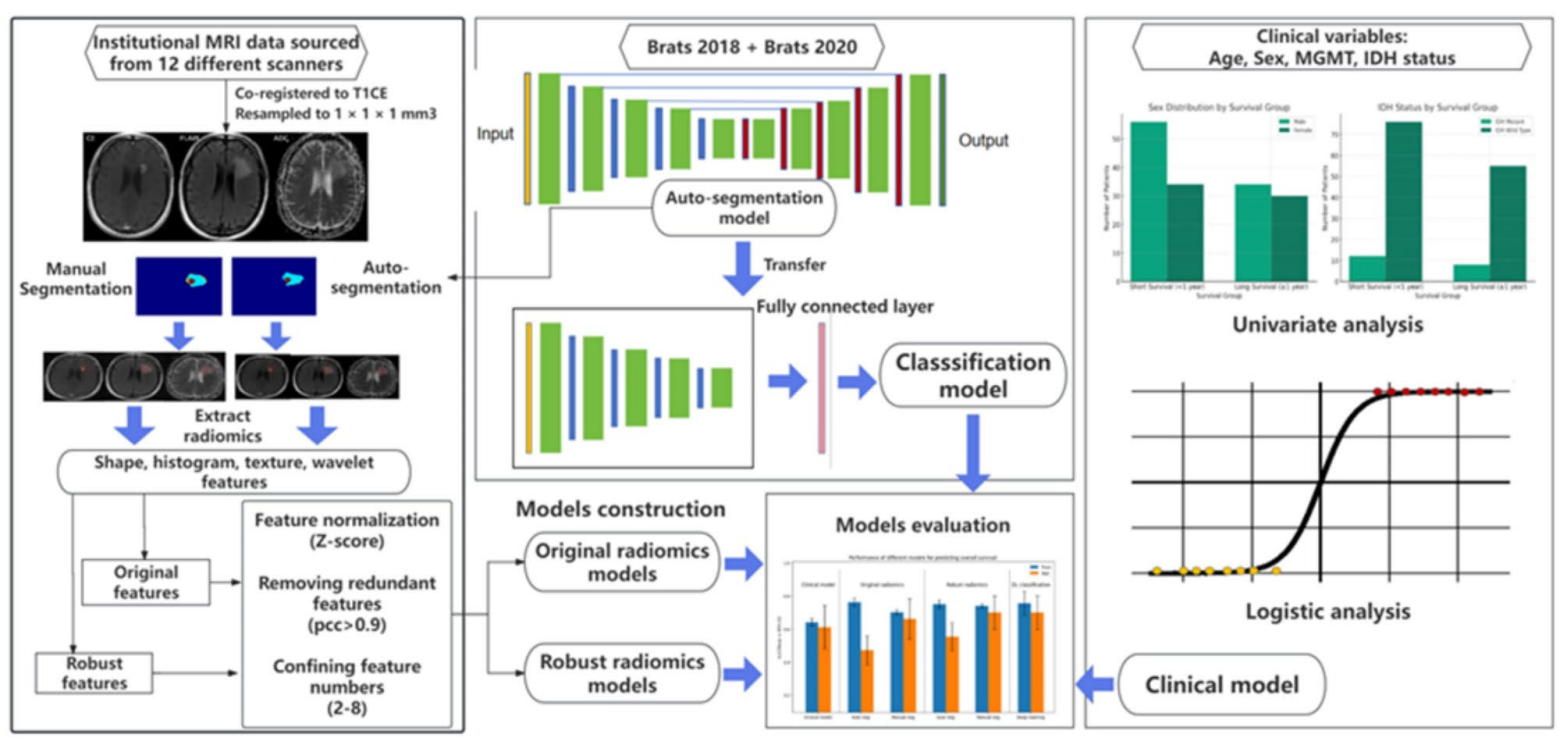
Workflow for our study. Institutional MRI data were processed and segmented using both manual and automated methods to extract radiomic features. A clinical model based on clinical variables, four radiomics models (two based on original radiomics features and the other two based on robust radiomics features), and a deep learning classification model were constructed and evaluated for predictive accuracy

Exclusion criteria include participants with intracranial conditions (e.g., epidural hematoma) causing brain tissue displacement or swelling(*n* = 2), which could affect assessment. Incomplete MRI data (e.g., missing T1-weighted contrast-enhanced images, *n* = 4); unsatisfying automated segmentation (*n* = 5). OS was defined as the time from immunotherapy initiation to death. For patients alive at the last follow-up, OS was censored at the time of last recorded contact. OS was dichotomized into intervals of less than 1 year, and 1 year or greater [9]. Figure 1 shows the flow chart of our study.

### Image acquisition and preprocessing

The MRI data retrieved included fluid-attenuated inversion recovery T2-weighted (FLAIR), Apparent Diffusion Coefficient (ADC) maps calculated from diffusion-weighted imaging, and post-contrast T1-weighted (T1CE) sequences. These images were sourced from 12 different scanners made by two vendors (GE Medical Systems^®^ and Siemens Healthineers^®^), at two institutions (Dana-Farber Cancer Institute and Brigham Women’s Hospital). To ensure consistency and comparability, all images were co-registered to the T1CE and interpolated to a uniform resolution of 1 × 1 × 1 mm³.

### Tumor segmentation

Manual segmentation was performed independently by two radiologists, with 10 and 7 years of experience respectively, using 3D Slicer^®^ software (Harvard Medical School, Boston, MA, USA, https://www.slicer.org). ROIs were delineated separately on T1CE, ADC, and FLAIR sequences to generate a comprehensive whole-tumor ROI, that comprised both the central enhancing and surrounding non-enhancing portions of the tumor.

For automated segmentation, we employed a SegResNet [10] based CNN architecture, optimized with the Adam optimizer and cosine annealing as the scheduler. The model was trained using a hybrid loss function, which combined DiceLoss and CrossEntropy Loss. The CNN was initially trained on the publicly available BraTS 2018 [11–13] (*n* = 210) and BraTS 2020 [11–13] (*n* = 369) datasets. To enhance the model’s performance and ensure it is effective for both newly diagnosed and recurrent HGG, we fine-tuned it using 100 randomly selected cases from our institution: 73 randomly selected from the 154 included recurrent HGG cases, augmented by 27 newly diagnosed HGG cases. The inclusion of both newly diagnosed and recurrent cases was driven by our goal to create a model robust enough for glioma segmentation across different stages of the disease.

The segmentation model used in this study was part of our previous research [8]. Details of the model’s architecture, initial training process and segmentation details, including DICE scores for each sequence and the overall subjective score, were reported in our previous paper [8].

### Deep learning model

For deep learning prognosis classification, we truncated the SegResNet, keeping only the encoder arm, while replacing the decoder arm with an integrated classification head composed of a convolutional layer followed by a dense layer with dimensions 128 × 1024. This modification was designed to facilitate the transition from feature extraction to classification, ensuring that the network effectively interprets and categorizes the extracted features.

### Radiomics analysis

**Fig. 2:**
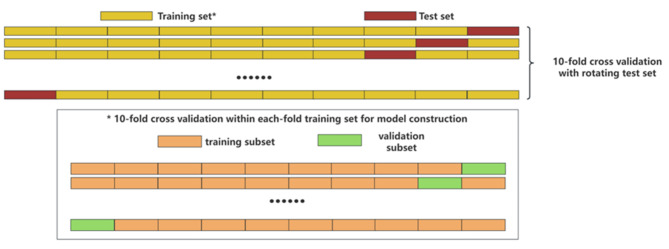
Schematic of data splitting and cross-validation strategy. To minimize bias, a stratified ten-fold cross-validation with a rotating test set was employed

The sample size ratio between the training set and the testing set was 9:1. Within the training set, we performed ten-fold cross-validation, meaning that in each fold, the training set was further divided into a smaller training subset and a validation subset. To minimize bias and ensure a robust evaluation of the models, we used a stratified ten-fold cross-validation with a rotating test set. This means that in each fold, a different subset of the data is used as the test set, while the remaining data is used for training and validation. This process ensures that every data point is used for testing exactly once. (Fig. 2)

We used Pyradiomics to extract a comprehensive set of features (*n* = 851) from each of the manually-segmented and CNN-segmented whole-tumor ROI, including shape, intensity, texture, and wavelet-based features, from each of the ADC, FLAIR, and T1CE. This yielded a total of 2553 (851 × 3) original features for each ROI. For cases with multifocal tumors, each lesion was labeled, and radiomic features were extracted from all lesions combined as a single dataset per patient.

After Z-score normalization, For each pair of features with a PCC > 0.90, one feature was randomly removed. Further feature selection was then performed using the Kruskal-Wallis (KW) test. Then the feature set was input into the support vector machine (SVM) classifier, and performance of data from each segmentation type was compared. Next, original features with ICC > 0.75 between manual and auto-segmented ROIs were selected as robust features, reducing the total number of included features to 1278 in each model. These robust features also underwent the same normalization and feature selection process and input into the SVM classifier and the performance of data from each segmentation type was compared. The whole process was repeated 10 times for different folds. FeAture Explorer [14] (FAE, V0.5.3, https://github.com/salan668/FAE) was used for all the analysis.

### Statistical analysis

The Kolmogorov-Smirnov test was used to assess the normality of data distribution. For normally distributed data, differences were evaluated with the independent sample t-test; for non-normally distributed data, the Mann-Whitney U test was used for group comparison. Cox regression analysis was performed to determine the association between clinical variables and the endpoint. We incorporated variables with *P* < 0.05 in the univariable Cox regression analysis into the multivariable analysis. Multivariate logistic regression was used to build a binary prognostic model. Receiver operating characteristic (ROC) curve analysis assessed the diagnostic performance of clinical, radiomics, and deep learning models. The area under the ROC curve (AUC) and its 95% confidence intervals (CIs) were calculated across ten data folds to ensure robustness. All statistical analyses were performed using Python (version 3.9). Statistical significance was defined as a p-value less than 0.05.

## Results

### Participant characteristics

The demographic and clinical characteristics of patients are shown in Table 1. In our cohort, there were 133 cases of IDH-wildtype GBM and 21 cases of other HGG, including 1 case of diffuse midline glioma (H3K27M-mutant), 18 cases of IDH-mutant Astrocytoma, and 1 case of IDH-mutant, 1p/19q co-deleted Oligodendroglioma. Patients with short survival had an average age of 57.78 years (± 12.13), while those with long survival were younger, averaging 53.27 years (± 12.61) (*P* = 0.028). There was a notable trend in O^6^-methylguanine-DNA methyltransferase (MGMT) promoter methylation status. In the short survival group, 46 (58.97%) patients had unmethylated, 6 (7.69%) partially methylated, and 26 (33.33%) methylated MGMT promoters. Conversely, in the long survival group, these numbers were 23 (38.33%), 7 (11.67%), and 30 (50.00%) respectively. The trend approached statistical significance (*P* = 0.056). Gender and IDH status did not show a significant correlation with OS (*P* = 0.259 and 0.867 respectively).

**Table 1:** Clinical characteristics of patients

Clinical features		Overall survival (%)		*P* value
		Short survival (< 1 year, *n* = 90)	Long survival (≥ 1 year, *n* = 64)	
Age		57.78 ± 12.13	53.27 ± 12.61	0.028*
Sex				0.259
	Male	56(62.22)	34(53.13)	
	Female	34(37.78)	30(46.88)	
IDH-status				0.867
	Mutant	12(13.33)	8(12.70)	
	Wild type	78(86.67)	55(87.30)	
MGMT promoter methylation				0.056
	Unmethylated	46(58.97)	23(38.33)	
	Partially methylated	6(7.69)	7(11.67)	
	Methylated	26(33.33)	30(50.00)	

* denotes *p* < 0.05

### Univariate and multivariate Cox regression

The univariate Cox regression analysis identified age(*p* = 0.039) and MGMT methylation(*p* = 0.035) are significant OS predictors in HGG patients. The multivariate Cox regression analysis revealed that the hazard ratio (HR) for age was 1.0178 (95% CI: 1.001–1.034, *p* = 0.037). Patients with methylated MGMT had a significantly reduced hazard of death (HR = 0.658, 95% CI: 0.444–0.975, *p* = 0.037) compared to those with unmethylated MGMT. However, the partially methylated MGMT group did not show a statistically significant difference in survival (HR = 0.730, 95% CI: 0.378–1.409, *p* = 0.348).

### Performance of clinical models

We included MGMT promoter methylation and age as predictors in our clinical model. The model achieved an AUC of 0.640 ± 0.013 in the training set and 0.610 ± 0.131 in the test set across 10 folds.

### Lesion segmentation

**Fig. 3:**
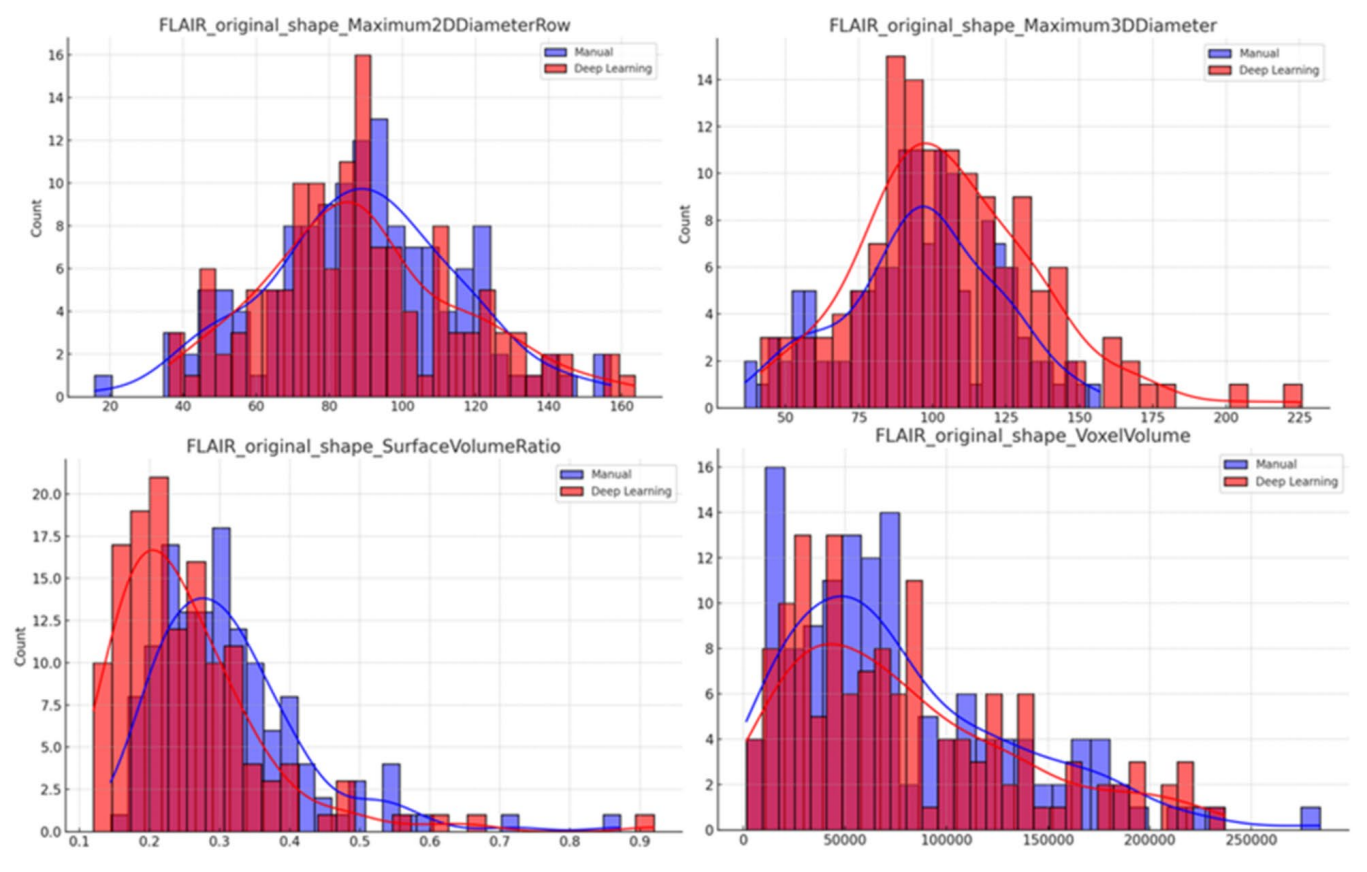
Distributions of a selection of diameter and volume-related features from both manual and deep learning segmentation methods. There is a considerable overlap between the blue (manual segmentation) and red (deep learning auto-segmentation) histograms suggesting that both segmentation methods yield fairly similar morphological features across the dataset

**Fig. 4:**
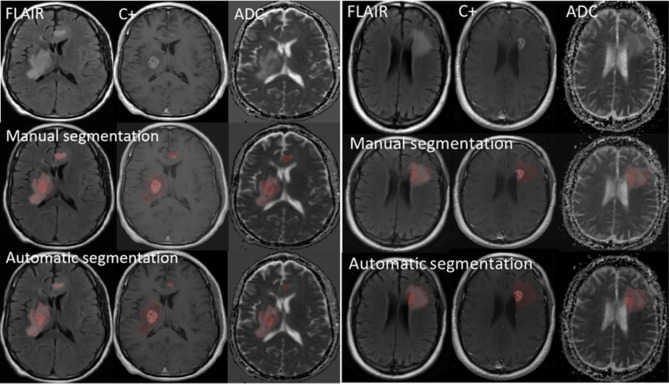
Manual and automatic segmentation examples in short overall survival (left) and long overall survival group (right). Top row: FLAIR, contrast-enhanced (C+), and ADC images. Middle row: Manual segmentation overlay on the respective images, with enhancing tumor highlighted in semi-transparent dark red. Bottom row: Automatic segmentation results using a CNN-based approach, depicted with the same color scheme

Figure 3 shows the distribution of diameter and volume-related features of manual and auto segmentation indicating a good consistency between the automated and manual methods. Figure 4 shows some examples of the comparison of the two segmented ROIs. All lesions were detected by CNN and all segmentations were checked by a radiologist with 10 years’ experience. Subjective reviews revealed no significant difference between automatic and manual segmentation [8]. 

### Performance of radiomics models

**Fig. 5:**
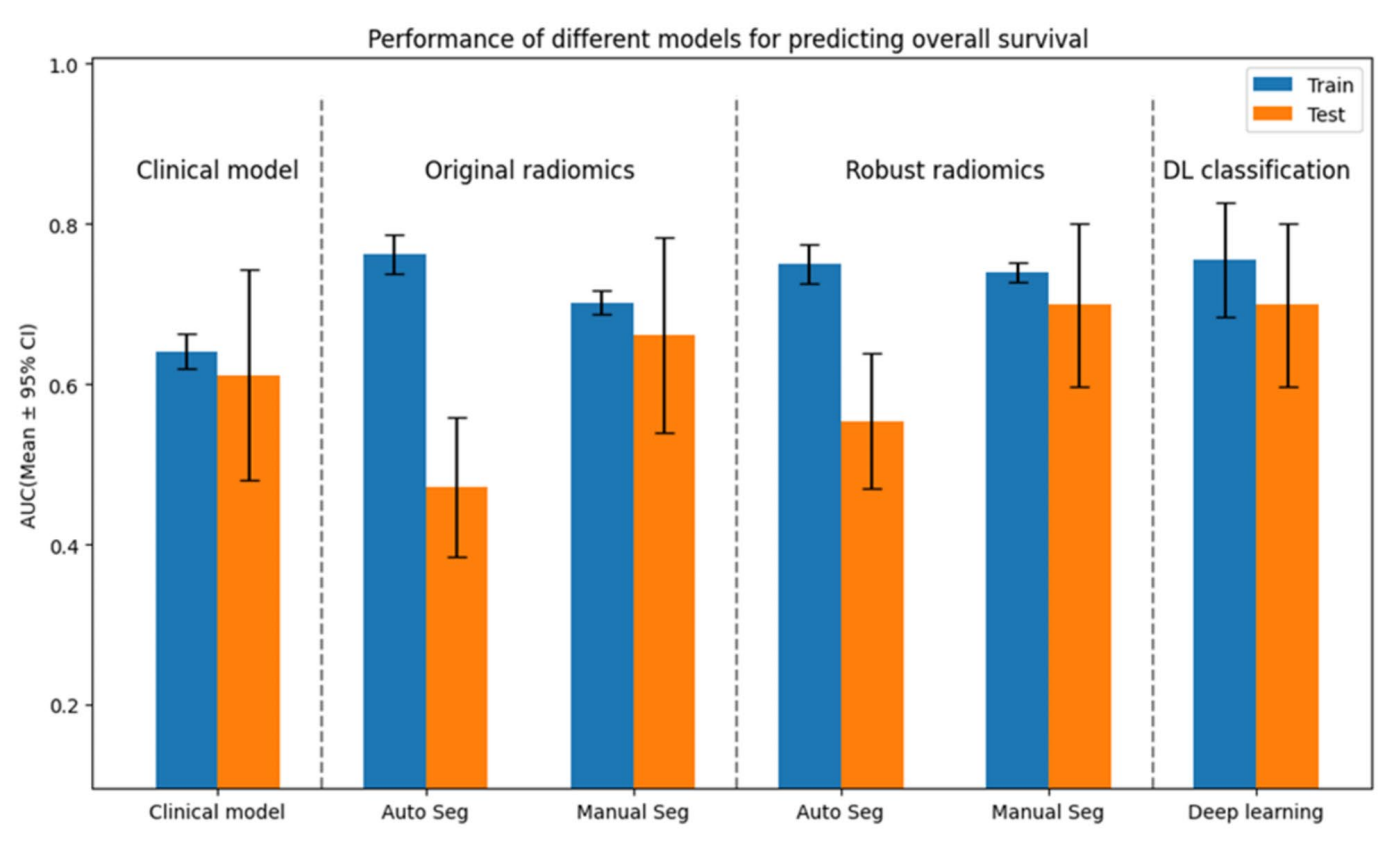
Performance of clinical, radiomics and deep learning models for predicting overall survival in recurrent high-grade glioma patients who underwent immunotherapy. Performance is measured by the area under the receiver operating characteristic curve with a 95% confidence interval, comparing training (blue) and test (orange) datasets. (Auto Seg: Automated segmentation, Manual Seg: Manual segmentation)

Figure 5 illustrates the performance of different radiomics models compared to clinical models and differentiated by segmentation technique. Models using features from manual segmentation achieved a mean AUC in the test set of 0.662 ± 0.122, compared to models using features from automatic segmentation that achieved a mean AUC of 0.471 ± 0.086 in the test set across 10 folds.

Models using only the 1287 robust features selected based on ICC > 0.75 demonstrated improved performance: manual segmentation mean AUC of 0.700 ± 0.102, CNN-segmentation mean AUC of 0.554 ± 0.085.

### Performance of deep learning models

The CNN-prognosis classification model achieved an average AUC of 0.755 ± 0.071 in the training set, and 0.700 ± 0.101 in the test set, across 10 folds.

## Discussion

Accurate prediction of survival may help select HGG patients for trials of novel immunotherapies or targeted molecular therapies in which variability in patient responses poses significant challenges, and can be helpful for personalizing HGG therapy in individual patients. We compare automated CNN-segmentation with manual-segmentation based radiomics OS prediction and clinical OS prediction model in patients with recurrent HGG treated with immunotherapy. Radiomics models using features extracted by expert manual segmentation yielded better performance than equivalent models using features extracted by automated segmentation. The performance of both radiomics models improved after applying robust features. The CNN-prognosis end-to-end classification model, combining an encoder-arm trained for tumor segmentation with a classifier trained to predict overall survival performed comparably to the radiomics model.

In this study, we demonstrate some important clinical prognostic factors in recurrent HGG patients treated with immunotherapy. Our findings suggest the significance of MGMT promoter methylation as a positive prognostic marker, aligning with a recent meta-analysis [15]. However, the partially methylated MGMT group did not show a statistically significant difference in survival. Conversely, advanced age correlates with reduced OS, potentially due to age-related immune suppression in the brain [16]. We developed clinical models aimed at predicting OS, which demonstrated moderate efficacy. This indicates that relying solely on clinical variables might offer limited predictive power in forecasting survival outcomes for recurrent HGG patients undergoing immunotherapy.

The application of radiomics and machine learning for imaging-based prognosis prediction in HGG patients undergoing immunotherapy remains under-explored. A previous study indicates that high PD-L1 levels, as predicted by these methods, are associated with a better prognosis [17]. A recent study demonstrated that a radiomics-based machine learning model, using first on-treatment MR imaging features, can predict survival rates in patients with glioblastoma undergoing PD-L1 inhibition immunotherapy [18]. Previous studies largely relied on manual segmentation, which is time-consuming and subject to inter-operator variations [19, 20]. We report the development and testing of two distinct CNN-based approaches to automate prognostic analysis: CNN-segmentation and end-to-end CNN-prognosis models, and compare them to OS prediction using manual segmentation-based radiomics and known clinical and molecular features. Our investigation revealed that, although the CNN segmentation was qualitatively similar to manual segmentation, predictive models based on manual segmentation by radiologists outperformed those using automated segmentation. It seems likely that the shape (diameter and volume) features and semi-quantitative analysis of the ROIs were not sufficiently sensitive to capture the finer distinctions that are pivotal for prognostic evaluations. On the one hand, this highlights an ongoing need for expert input, especially in complex scenarios like postoperative cases where automated segmentation is often challenging [21, 22]. It illustrates that more work is needed to fully automate radiomics-based modeling in reccurrent HGG.

Our finding that the selection of robust feature subsets substantially improved radiomics model performance suggests that this strategy deserved further research. In the context of GBM MRI radiomics, studies have investigated the robustness of radiomic features across various image preprocessing methods, registration algorithms, and segmentations by different experts [23–26]. Our results demonstrated that the identification of robust features shared between manual and automated segmentation improved OS prediction. Our findings align with prior research showing that selecting stable features enhances the accuracy of prognostic models compared to those trained with less robust features [27]. This raises the question of whether a subset of radiomics features could be identified that would yield more consistent results across a wide range of different datasets.

Previous studies have introduced deep learning models as a promising tool for predicting the prognosis of glioblastoma [28, 29]. The majority of these studies built their prediction models based on tumor segmentation or cropped image regions that contain only the tumor, neglecting information available in the surrounding tissue [30]. This may omit crucial prognostic data, as highlighted by recent research indicating that sarcopenia metrics derived from temporalis muscle, as analyzed by a DL model, correlate with GBM outcomes [31]. This underscores the potential prognostic value embedded within non-tumoral regions. Our results are consistent with this, demonstrating promising results for recurrent HGG prognosis prediction using a modified CNN-prognosis model that extracts features from the whole brain, circumventing the need for specific tumor delineation.

There are several limitations in our study. First, although the heterogeneity of our image data, comprising 12 scanners, 2 vendors and 2 institutions, should help with generalizability, our sample size is relatively small. To mitigate the small sample size, we employed a 10-fold cross-validation approach with a rotating test set. Validation of these results in larger and more diverse patient cohorts will be essential. Second, in this study, radiomics analysis was performed based on the whole tumor segmentation rather than on separate masks. The primary reason for this choice was that whole tumor segmentation showed the highest similarity between manual and CNN segmentation. To avoid issues related to segmentation quality, we opted for whole tumor analysis. Future research should explore the impact of radiomic features derived from individual sequences on predictive performance. Third, while our radiomics and deep learning models predict prognosis in recurrent HGG patients receiving immunotherapy, the lack of a non-immunotherapy control group limits the attribution of outcomes specifically to immunotherapy. Additionally, concurrent treatments introduce variability in effects. Our findings serve as a preliminary step towards identifying immunotherapy-specific prognostic markers, though further research with control groups and standardized treatments is needed to validate these markers. Fourth, the choice of machine learning methods could impact the results of predictive modeling [32]. In this study, we used SVM, as it is a relatively common and generally well-performing method. However, exploring different machine learning methods for predicting HGG prognosis may be beneficial in future studies. Finally, interpretability remains a major challenge for CNN and remains a major barrier to the clinical translation of these methods.

In conclusion, our study demonstrates that CNN-based brain tumor MRI segmentation has the potential as a tool to facilitate research in HGG patients, but is not yet ready to fully replace human-expert annotation in complex scenarios such as postoperative cases. Identification of robust radiomic feature subsets can enhance radiomics model performance and deserve further investigation. End-to-end, segmentation-independent, CNN prediction of prognosis, demonstrated comparable performance to radiomics models in this small dataset, but at the cost of posing greater problems for interpretability.

## Data Availability

The data are not publicly available due to privacy or ethical restrictions.

## Abbreviations

AUC: area under the curve
CNN: convolutional neural networks
DL: deep learning
OS: overall survival
SVM: Support Vector Machine
GBM: glioblastoma
MGMT: O6-methylguanine-DNA methyltransferase
IDH: isocitrate dehydrogenase
HGG: high grade glioma
